# Signatures of Natural Selection at the *FTO* (Fat Mass and Obesity Associated) Locus in Human Populations

**DOI:** 10.1371/journal.pone.0117093

**Published:** 2015-02-03

**Authors:** Xuanshi Liu, Kerstin Weidle, Kristin Schröck, Anke Tönjes, Dorit Schleinitz, Jana Breitfeld, Michael Stumvoll, Yvonne Böttcher, Torsten Schöneberg, Peter Kovacs

**Affiliations:** 1 IFB AdiposityDiseases, University of Leipzig, Leipzig, Germany; 2 Bioinformatics Group, Department of Computer Science, University of Leipzig, Leipzig, Germany; 3 Department of Medicine, University of Leipzig, Leipzig, Germany; 4 Institute of Biochemistry, Department of Medicine, University of Leipzig, Leipzig, Germany; Cincinnati Children’s Hospital Medical Center, UNITED STATES

## Abstract

**Background and aims:**

Polymorphisms in the first intron of *FTO* have been robustly replicated for associations with obesity. In the Sorbs, a Slavic population resident in Germany, the strongest effect on body mass index (BMI) was found for a variant in the third intron of *FTO* (rs17818902). Since this may indicate population specific effects of *FTO* variants, we initiated studies testing *FTO* for signatures of selection in vertebrate species and human populations.

**Methods:**

First, we analyzed the coding region of 35 vertebrate *FTO* orthologs with Phylogenetic Analysis by Maximum Likelihood (PAML, ω = dN/dS) to screen for signatures of selection among species. Second, we investigated human population (Europeans/CEU, Yoruba/YRI, Chinese/CHB, Japanese/JPT, Sorbs) SNP data for footprints of selection using DnaSP version 4.5 and the Haplotter/PhaseII. Finally, using ConSite we compared transcription factor (TF) binding sites at sequences harbouring *FTO* SNPs in intron three.

**Results:**

PAML analyses revealed strong conservation in coding region of *FTO* (ω<1). Sliding-window results from population genetic analyses provided highly significant (p<0.001) signatures for balancing selection specifically in the third intron (e.g. Tajima’s D in Sorbs = 2.77). We observed several alterations in TF binding sites, e.g. *TCF3* binding site introduced by the rs17818902 minor allele.

**Conclusion:**

Population genetic analysis revealed signatures of balancing selection at the *FTO* locus with a prominent signal in intron three, a genomic region with strong association with BMI in the Sorbs. Our data support the hypothesis that genes associated with obesity may have been under evolutionary selective pressure.

## Introduction

Obesity is a complex disease with an estimated heritability of 40–70% [1,2] The existence of genetic factors is well supported by a number of polymorphisms identified in recent genome-wide association studies [3]. Single nucleotide polymorphisms (SNPs) in the fat mass and obesity-associated gene (*FTO*) locus seem to be among the eminent factors associated with obesity measures such as body mass index (BMI). The associations between *FTO* variants and BMI have been robustly replicated in populations with different ethnic backgrounds [4–7]. The human *FTO* maps on 16q12.2 and encodes a 2-oxoglutarate-dependent nucleic acid demethylase [8]. The SNP rs9939609 representing a cluster of variants with strong associations to BMI and overweight is located in the first intron [5]. Whereas these associations have initially been shown in cohorts of European origin [5], they could not be replicated in an African sample and the Han Chinese [9,10]. On the other hand, in the Sorbs—a population of Slavic origin residing in Eastern Germany, in addition to the association signal in the first intron, the strongest association to BMI was found for the SNPs mapping to intron three [11]. These findings indicate specific effects of *FTO* alleles in Sorbs and raise the question, whether *FTO* has been subject to natural selection and so, does show population specific patterns of selection.

Considering the “thrifty genotype” hypothesis which states that an evolutionarily advantageous increased capacity to store energy may result in obesity and type 2 diabetes (T2D) in Western-lifestyle societies [12], genes associated with obesity and T2D have become attractive targets of evolutionary studies. Until recently, there has not been a strong evidence for consistent patterns of selection at loci associated with T2D which would provide conclusive confirmation of the thrifty genotype hypothesis. It has been shown more recently that in a locus-by-locus study, 14 loci associated with T2D, and to a lesser extent, obesity, from European, Africans and East Asian populations appear to have undergone selection, however there is no positive selection evidence found when all the T2D loci were analyzed together [13].

Since comprehensive data regarding *FTO* evolution are sparse, we initiated studies searching for signatures of selection in vertebrates and human populations. To test for the conservation of the protein-coding sequence on the inter-species level, we calculated the ratio of non-synonymous to synonymous base exchanges (ω = d_N_/d_S_) of the coding region in 35 vertebrate *FTO* orthologs with Phylogenetic Analysis by Maximum Likelihood (PAML). Furthermore, we investigated SNP data for footprints of selection using DnaSP version 4.5 and the Haplotter/PhaseII in human population of Yoruba from Ibadan, Nigeria (YRI), Utah residents with Northern and Western European ancestry (CEU), East Asians, (ASN), Han Chinese from Beijing, China (CHB), and Japanese from Tokyo, Japan (JPT)). Finally, we examined *in silico* the possible impact of *FTO* SNPs in intron 3 on transcription factor (TF) binding sites.

## Materials and Methods

All studies were approved by the ethics committee of the University of Leipzig. All subjects gave written informed consent.

### Phylogenetic Analyses by Maximun Likelihood (PAML)

**Figure 1:**
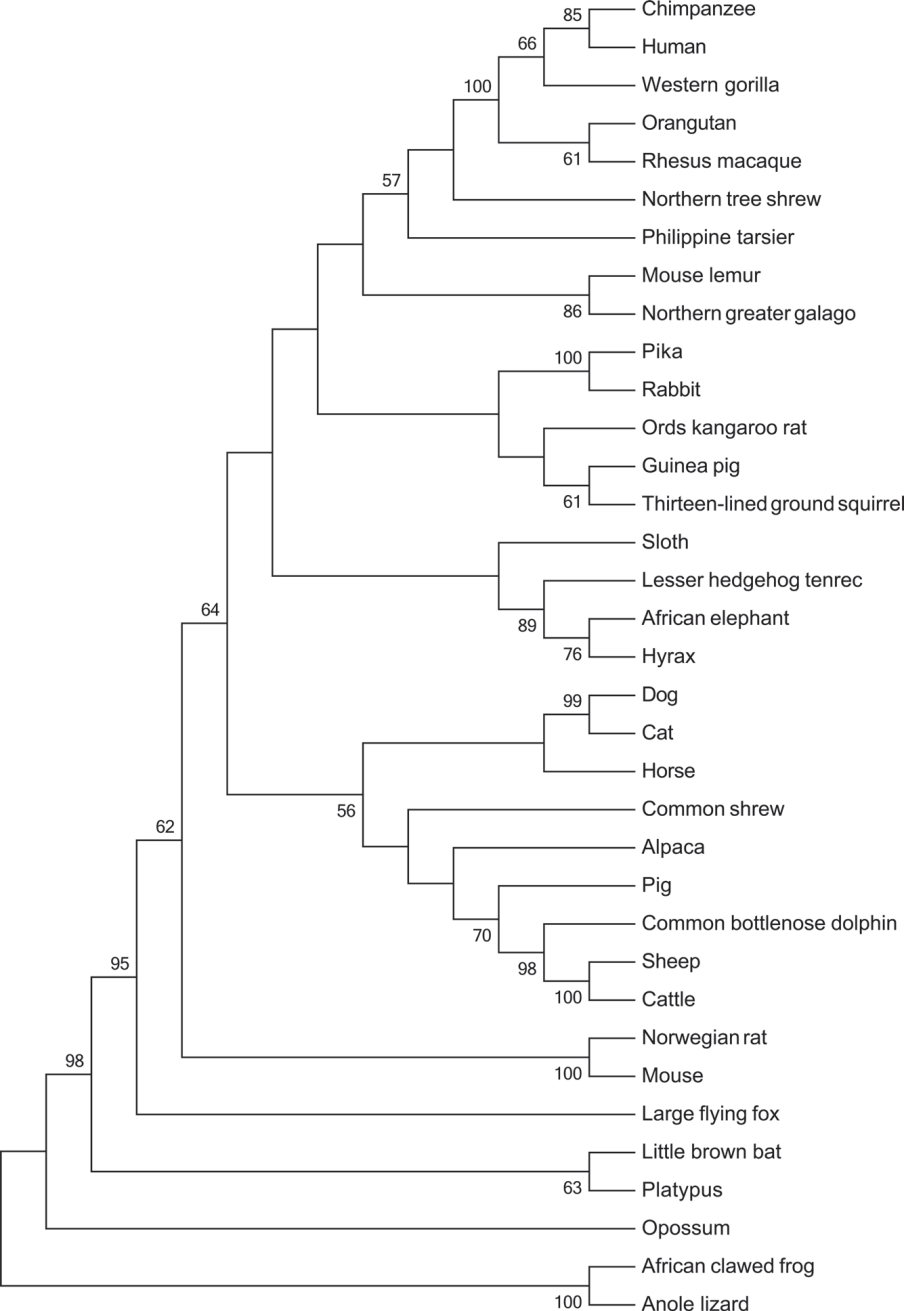
Neighbor-joining phylogenetic tree of 35 species on *FTO* coding sequences showing the evolutionary relationship. The bootstrap consensus tree inferred from 1000 replicates is generated to present the evolutionary relationship among 35 species on *FTO* coding sequences which were retrieved from Ensembl or NCBI. Accession numbers are listed at S1 Table. Alignment was carried out by ClustalW (1581 nucleotides left) and phylogenetic tree is constructed by neighbor-joining method in MEGA6. Branches corresponding to partitions reproduced in less than 50% bootstrap replicates did no display. This tree works as the initial tree for further PAML analysis.

PAML [14] provides a program CODEML to estimate the level of gene conservation by calculating the d_N_/d_S_ ratio ω (d_N_: non-synonymous mutation substitution rate, d_S_: synonymous mutation substitution rate). In the present study, ω was calculated in PAML version 4.1 [15]. The coding sequences of 35 vertebrate *FTO* orthologs were extracted from Ensembl (http://www.ensembl.org) and the NCBI (http://www.ncbi.nlm.nih.gov) databases. Species and accession numbers are provided in S1 Table. Subsequently, all coding sequences were aligned by a widely used progressive alignment method, ClustalW [16] within MEGA 6 [17]. Phylogenetic tree was conducted by Neighbor-Joining (NJ) algorithm using aligned coding sequences in MEGA 6. The evolutionary distances were computed by Jukes-Cantor model which is the best predicted model giving by jModeltest 2.1.5 [18]. 1,000 bootstrap searches were performed to infer the phylogenetic tree and bootstrap consensus phylogenetic tree. The initial input for PAML analysis is displayed in Fig. 1.

Due to the fact that the power to detect positive selection is reduced when the rates across sites are averaged, diverse tests were adopted according to recommendations for real data analyses [19]. The tests conducted include the one-ratio model (M0), free-ratio [20], nearly neutral (M1a), positive selection (M2a) [21], discrete (M3) [22], beta (M7), and beta&ω (M8) [23]. The likelihood ratio tests (LRT = 2 (*l*_1_–*l*_0_), 2Δl, where *l*_1_ and *l*_0_ are the log likelihoods from two models respectively) are conducted to every two nested models [24] in order to identify which model better fits to the data. LRTs and nested models are briefly introduced as followed: the M0 model is a plain model in which the same d_N_/d_S_ ratio is assumed for all branches in the phylogeny [20]. The free-ratio model is the most general model, where an independent d_N_/d_S_ ratio is assumed for every branch [20]. The first LRT involves the M0 model and the free-ratio model which can be compared to survey if the d_N_/d_S_ ratios ω differ among lineages [20]. Paired M0 model and M3 models can be tested by the second LRT, which is to analyze if ω varies among sites. In the discrete model, M3, three site classes, each of them with an independently estimated ω which also allows for sites with ω > 1, are estimated over a general discrete distribution. For each site class the proportion *p* is given [22]. The third LRT compares the M1a model and M2a model. M1a postulates a class of sites with ω = 0 and a second class of sites with 0 < ω < 1, where in the M2a model a third class of sites is added (ω >1) [25,24]. The fourth LRT is between M7 model and M8 model, which has more power to detect positively selected sites, as both models allow for sites with 0 < ω < 1 [23]. For M7 a beta distribution for ω over sites is assumed, which is limited to the interval (0, 1) [23]. In M8 another site class is added with ω valuated from the data set which allows sites with ω > 1 [23].

### SNPs selection and population genetic measures

Population genetic measures were calculated with DnaSP version 4.50.0.3 [26], Sweep [27], and the iHS (integrated haplotype score) tool available under http://hgdp.uchicago.edu/. In the Sorbs cohort, a self-contained ethnic group in Eastern Germany, which has been described in detail elsewhere [11], closely related subjects (Identity by descent/IBD > 0.05, calculated by PLINK v1.01 [28] ) were removed from the analyses. Further inclusion criteria were: minor allele frequency (MAF) > 0.01, Hardy-Weinberg equilibrium (HWE) > 0.0001, missingness per SNP < 0.05, and missingness per sample < 0.07. According to these standards, genotype data for 307 SNPs within the *FTO* locus (positions ranging from 52013417 to 52997859 bp, NCBI build 35/hg17) in 34 individuals were extracted from an available dataset obtained by genotyping DNA from about 1000 Sorbs with 500K/6.0 Affymetrix GeneChip arrays (S2 Table).

Additionally, data of the HapMap populations (YRI, CEU, ASN, CHB, JPT) were downloaded from http://www.hapmap.org/ and filtered for the same SNPs genotyped in the Sorbs (S2 Table). As the CEU and YRI comprise parent/child trios, analyses were performed without SNP data of the children. Haplotype reconstruction in all populations was performed with PHASE version 2.1 [29,30].

DnaSP provides population genetic measures like Tajima’s D [31], Fu and Li’s D*, and Fu and Li’s F* [32], which all detect deviations from the normal distribution of common or rare alleles in neutral evolution. The iHS is based on different levels of linkage disequilibrium (LD) surrounding selected allele region compared to the background allele at the same position. Suggestive evidence for natural selection is defined as iHS < −1.5 or > 1.5, powerful selection is iHS < −2 or >2 [33]. The calculated fixation index (Fst) is a measure for the extent of variations in allele frequency between populations [34]. Population differentiation increased by local adaptation may result in larger Fst values [35].

For the HapMap populations, standardized iHS and Fst values were also provided by the Haplotter (http://hg-wen.uchicago.edu/selection/haplotter.htm.) [36,37]

### Transcription factor binding sites

To uncover alleles that may change binding sites in intron three, sequences surrounding eight obesity associated SNPs (20bp up—and downstream) within *FTO* intron three were downloaded from UCSC genome browser (http://genome.ucsc.edu/). Comparing transcription factor binding probabilities of sequences carrying either the major or the minor alleles were performed using ConSite [38]. Sequences included in the analysis are listed in S4 Table. We particularly analyzed transcription factors specifically in vertebrates, as it is well acknowledged that functional transcription factor binding sites are conserved among close species, where substitutions occur mostly at nonfunctional positions when the evolutionary distance of species increases [39]. ConSite incorporates the datasets from JASPAR [40] and ConSite summarizing transcription factor binding profiles as well as phylogenetic footprinting algorithms for additional constraints further improving prediction algorithms. The noise level of ConSite compared to other single sequence analysis is reduced by ∼ 85% [38]. The program has been applied to several studies [41–43] and has been validated in functional experiments both *in vitro* and *in vivo* [42].

## Results

### Phylogenetic Analyses by Maximum Likelihood (PAML)

PAML analysis revealed that the coding region of *FTO* is highly conserved among all studied species (average ω = 0.1616). The LRT statistic for lineage-specificity model (M0 *vs*. free-ratio) was calculated as 2Δl = 63.74. Compared with a χ^2^ distribution under d.f. = 66, the difference between these two models was not significant indicating that the ω is not different among lineages. This suggests no differences in the direction and magnitude of selection acting on *FTO* coding regions of each species [44]. The second LRT was conducted between M0 model and M3 model. The significance of the result of LRT (2Δl = 193.47, d.f. = 4 ) pointed to the M3 model. In this case, ω varied among sites within a species instead of having a constant value, and the substitution rate between non-synonymous and synonymous mutations fluctuated within *FTO* coding region of each species. In the last two LRTs, non-significant results were detected which suggested null neutral hypothesis (M1a and M7) [45]. In summary, positive selection cannot be inferred for any of the sites in coding sequences. All data are summarized in Table 1.

**Table 1:** PAML analysis of *FTO*.

**Model**	**Parameter Estimates**	**In L**	**LRT**			
			**Models**	**2Δl**	***d.f.***	**p-value**
M0	ω_0_ = 0.1616	−1462.43	M0 *vs. free-ratio*	63.74	66	0.58
*free-ratio*	variable	−1430.56				
M3	p_0_ = 0.40765, p_1_ = 0.39476, p_2_ = 0.19759, ω_0_ = 0.00264. ω_1_ = 0.12330, ω_2_ = 0.90116	−1365.70	M0 *vs*. M3	193.47	4	< 0.001
M1a	p_0_ = 0.79894, p_1_ = 0.20106, ω_0_ = 0.05695, ω_1_ = 1.00000	−1378.39	M1a *vs*. M2a	0.61	2	0.74
M2a	p_0_ = 0.79860, p_1_ = 0.16914, p_2_ = 0.03227, ω_0_ = 0.06053, ω_1_ = 1.00000, ω_2_ = 1.90635	−1378.09				
M7	p = 0.21277, q = 0.75616	−1367.96	M7 *vs*. M8	3.71	2	0.22
M8	p_0_ = 0.94790, p = 0.26173, q = 1.37577 (p_1_ = 0.05210), w = 1.63117	−1366.10				

All values computed under default settings. InL: log likelihood, LRT = likelihood ratio test to detect positive selection, *d.f*. = degrees of freedom.

### Population genetic measures

**Figure 2:**
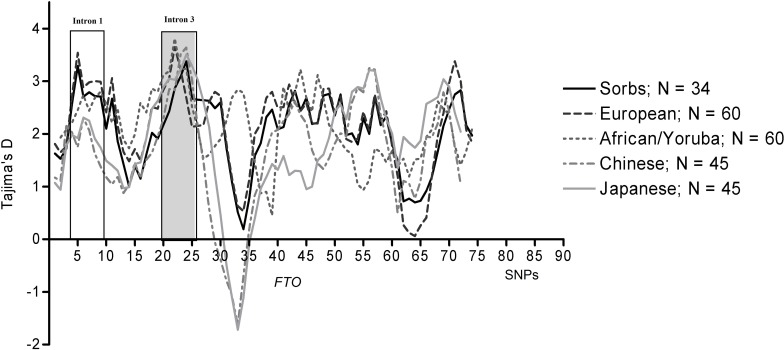
Tajima’s D within the *FTO* locus from five populations showing signals of selection. Sliding window analysis of Tajima’s D in the whole gene locus (∼ 1 Mb) was conducted with DnaSP version 4.50.0.3. Populations of European, African, Chinese, Japanese and Sorbs were included in the analysis. Closely related individuals were removed, i.e. trios from Chinese and Japanese populations and individuals with IBD > 0.05 from Sorbs. Higher values of Tajima’s D were observed at the intron 3.

The analyses with DnaSP provided strong evidence for a non-neutral evolution of the *FTO* locus. Across the whole gene locus (1 Mb), Tajima’s D showed significant deviations from the normal distribution of alleles (summarized in Table 2). The sliding-window analyses further supported these findings (Fig. 2). Interestingly, Tajima’s D seemed to be slightly higher in the third intron than in the first intron. Furthermore, the values across the studied populations in the third intron were more consistent when compared with the first intron which showed decreased Tajima’s D in Asian populations (Japanese and Chinese; Table 2 and Fig. 2). In line with Tajima’s D, also Fu and Li’s D* and Fu and Li’s F* tests showed significant deviations from neutrality in the investigated populations (Table 2).

**Table 2:** Results of the DnaSP Analyses.

**Data**	**n**	**SNPs**	**Tajima’s D**	***p***	Fu and Li’s D*	***p***	Fu and Li’s F*	***p***
***FTO***								
CEU	120	155	2.8228	**	2.4349	**	3.1269	**
CHB	90	152	2.173	*	2.0845	**	2.5467	**
JPT	90	152	2.3878	*	1.7632	**	2.4324	**
Sorbs	68	155	2.605	*	2.1099	**	2.7689	**
YRI	120	147	2.8541	**	2.5895	**	3.2467	**
**Intron 1**								
CEU	120	31	2.76787	**	1.30288	^#^	2.26319	**
CHB	90	30	1.8609	^#^	1.91378	**	2.24036	**
JPT	90	30	1.90727	^#^	1.91378	**	2.28686	**
Sorbs	68	31	2.48058	*	1.32095	^#^	2.09295	**
YRI	120	27	2.67329	*	1.88863	**	2.63200	**
**Intron 3**								
CEU	120	12	3.378	**	1.4607	^#^	2.5645	**
CHB	90	11	2.7264	**	1.4261	^#^	2.2333	**
JPT	90	11	3.0817	**	1.4261	^#^	2.3809	**
Sorbs	68	12	2.7653	**	1.4762	^#^	2.275	**
YRI	120	12	3.4569	***	1.4607	^#^	2.5984	**

n = number of haplotypes

* *p*<0,05

** *p*<0,02

*** *p*<0,001

^#^ 0,1>*p*>0,05

*FTO*: 52.297.274–52.696.065 bp; rs1421091 - rs2689269 on Human May 2004 (NCBI35/hg17), Intron 1: 52.326.794–52401034bp; rs7203521 - rs6499646, Intron 3: 52.421.901–52.434.067 bp; rs7204916 - rs7205213

From the publicly available data, the Haplotter showed iHS top scores > 2 in the CEU on the *FTO*-region, e.g. for rs7193144 and rs8050136 (S3 Table). All SNPs in the third intron had rather low iHS-values. These results were not significant according to the published map of recent positive selection in the human genome [36]. The unstandardized iHS values calculated with the iHS-tool supported publically available data in the Haplotter (Table 3 and S3 Table). It is noteworthy that in the Sorbs, the iHS for SNPs in the third intron (rs17818902 and rs17818920) was nearly three times higher than in the CEU sample (1.468 vs. 0.590). Notably, the strength of association with BMI positively correlated with the unstandardized iHS Table 3. iHS values indicate that no long haplotype was observed for variants in *FTO*. The Fst values between comparisons were close to zero among variants which indicated no significant population differences (Table 3).

**Table 3:** Population genetic measures on unstandardized iHS and Fst.

			**Unstandardized iHS**					**Fst**				***p*/beta**
**SNP**	***p* (BMI)**	**Intron**	**Sorbs**	**CEU**	**CHB**	**JPT**	**YRI**	**CEU vs. Sorbs**	**CHB vs. Sorbs**	**JPT vs. Sorbs**	**YRI vs. Sorbs**	
rs1861869	0.01465	1	−0.294	−0.766	0.212	−0.839	0.261	0.0040	0.1283	0.0919	0.0081	0.068/−0.501
rs1861868	0.00938	1	−0.869	−1.029	−0.049	−1.005	−0.310	0.0017	0.0912	0.0592	0.0558	
rs9940700	0.07348	1	0.000	0.539	−0.618	−0.401	−0.693	0.0021	0.4219	0.3889	0.2070	
rs9939973	0.005447	1	−0.667	−1.411	−0.865	−0.664	−0.429	0.0059	0.0751	0.0543	0.0062	
rs9940128	0.01583	1	−0.667	−1.411	−0.866	−0.665	−0.429	0.0059	0.0751	0.0543	0.0062	
rs9922047	0.01035	1	−0.400	0.220	−0.876	−1.006	−1.171	0.0063	0.0034	0.0034	0.0781	
rs16952522	0.1976	1	n. a.	n. a.	−1.994	−1.746	n. a.	0.0020	0.0016	0.0150	0.0055	
rs17817288	0.006795	1	0.274	−0.500	0.294	0.534	−0.537	0.0060	0.0067	0.0064	0.0135	
rs1477196	0.02183	1	−1.197	−1.057	−1.701	−1.891	n. a.	0.0045	0.0053	0.0053	0.1362	
rs1121980	0.004003	1	0.529	1.179	0.197	−0.109	0.370	0.0059	0.0751	0.0543	0.0059	
rs7193144	0.003387	1	0.826	1.550	0.727	0.649	0.740	0.0053	0.1005	0.0664	0.0063	
rs16945088	0.5796	1	−1.989	0.220	−1.380	−1.338	−0.914	0.0061	0.0064	0.0062	0.0924	
rs8050136	0.003092	1	0.747	1.361	0.741	0.667	0.280	0.0047	0.1005	0.0664	0.0032	
rs9939609	0.008727	1	0.375	1.361	0.411	0.088	−0.019	0.0047	0.1005	0.0664	0.0049	
rs9930506	0.02465	1	0.748	1.220	0.741	0.667	−0.133	0.0059	0.0751	0.0482	0.0789	
rs11075994	0.9537	2	0.912	0.772	0.149	0.493	0.842	0.0043	0.0041	0.0201	0.0332	0.041/−0.959
rs1421090	0.1254	2	−0.598	−0.309	−0.916	0.103	−0.480	0.0132	0.1470	0.1807	0.0276	
rs9972717	0.7354	2	−1.106	−1.648	n. a.	n. a.	n. a.	0.0050	0.0761	0.0522	0.1063	
rs10852522	0.001739	2	0.146	0.199	0.111	0.138	0.896	0.0054	0.0456	0.0037	0.0053	
rs10521308	0.7355	3	n. a.	n. a.	−0.943	−1.439	−1.072	0.0174	0.0387	0.0387	0.0061	0.009/0.881
rs17818902	0.000632	3	1.468	0.590	1.101	0.915	0.888	0.0154	0.0011	0.0015	0.0598	
rs17818920	0.0007213	3	1.468	0.590	1.101	0.915	0.888	0.0154	0.0011	0.0015	0.0598	
rs8053367	0.0009839	3	−0.760	−0.348	−0.427	−0.348	0.080	0.0029	0.0062	0.0006	0.0029	
rs8053740	0.001025	3	−0.760	−0.348	−0.427	−0.348	0.080	0.0029	0.0062	0.0006	0.0029	
rs7203051	0.001116	3	−0.760	−0.348	−0.427	−0.348	0.138	0.0029	0.0062	0.0006	0.0038	
rs7205009	0.001319	3	−0.760	−0.348	−0.427	−0.348	0.073	0.0029	0.0062	0.0006	0.0029	
rs7205213	0.001292	3	−0.871	−0.348	−0.460	−0.399	0.073	0.0009	0.0067	0.0011	0.0044	

*p* = *p*-value for association to BMI in the Sorbs; iHS = integrated Haplotype score; CEU = Central Europeans; CHB = Han Chinese from Beijing; JPT = Japanese from Tokyo; YRI = Yoruba from Ibadan; n. a. = not available

### Transcription factor binding sites

To elucidate the potential functional mechanisms underlying the strong association of variants in *FTO’s* third intron with BMI in the Sorbs, we investigated *in silico* the impact of SNPs on predicting putative transcription factor binding sites (S5 Table). As shown in S5 Table, minor alleles at variants rs17818902 showing the top association signal with BMI in the Sorbs [11] and rs8053740 predicted novel binding sites for two transcription factors TCF3 and SOX17, respectively.

Further, at rs8053367 the presence of the minor allele led to binding of multiple transcription factors, namely FREAC-2, HNF-3beta, HFH-1, HFH-2 (S5 and S6 Tables), while the binding site of transcription factor Irf-1 seemed to be significantly compromised. Finally, minor alleles of rs17818920 and rs7205213 led to alterations in binding sites for HLF, SOX17 and HNF-3beta (S5 and S6 Tables).

## Discussion

Polymorphisms in the *FTO* gene have been shown to be associated with obesity in different ethnic groups of European and other ancestries [3–5,7,11,46]. Whereas associations with SNPs in the first intron have been robustly replicated, in the Sorbs, the strongest effects on BMI were found for variants in the third intron [11]. To address specific associations of *FTO* variants in Sorbs, we aimed to test the gene for signatures of selection in mammals and particularly in human populations.

PAML-analyses on *FTO* coding sequence from 35 vertebrates revealed constant results with ω < 1. In the NearlyNeutral (M1a) model, most sites in the coding sequence were under strong (∼80%) purifying selection or neutral mutation (∼20%) and experienced a very high rate of synonymous substitutions, thus suggesting strong gene conservation. This underlines the biological importance of the gene, as functionally relevant genes are expected to be highly conserved and thus subject to purifying selection [47]. The fact that *FTO* is subject to purifying selection is consistent with findings of Ohashi *et al*. who studied the genetic architecture of *FTO* polymorphisms in oceanic populations [48]. However, considering purifying selection being an important means of evolution to maintain the optimized form of a gene, it cannot be excluded that *FTO* variants were positively selected in the past when the ability to store energy was beneficial.

It is of note that PAML analysis only examined the coding sequence, however most of the obesity-associated SNPs, like rs9939609 and rs17818920, map in the intronic regions. Therefore, test statistics such as iHS and Fst which are independent from coding regions are inevitable in evolutionary analyses. It has been stated before that at least in the oceanic populations *FTO* does not seem to comply with the thrifty genotype hypothesis [48]. The analyses in these populations have only considered polymorphisms in the first intron. In the context of our present data, further studies systematically targeting the *FTO* locus in populations of different ethnic backgrounds will be inevitable. As we show in the present study, neither iHS nor Fst values indicate positive selection for any allele from the third intron in individual groups or groups of populations (see S1 Fig.). Thus, consistent with studies in oceanic populations, our data would not support the thrifty genotype hypothesis. In contrast, other population genetic measures like Tajima’s D, Fu and Li’s D*, and Fu and Li’s F* suggest the signature of balancing selection in *FTO* on a significant level. Detection of balancing selection might be explained by the fact that whereas Tajima’s D considers the sites themselves in terms of allele frequencies, it does not take into account the surrounding regions of sites through addressing LD (such as iHS) [49,50]. This is interesting when considering that polymorphisms in the third intron showed the strongest association with BMI in the Sorbs from Germany [11]. Remarkably, in the first intron, Tajima’s D is rather low in Asian populations when compared with European Caucasians, which might at least in part explain ethnic specificity in the genotype-phenotype associations with SNPs in this gene region. However, it has to be noted that Tajima’s D was consistent across studied populations in the third intron, which does not seem to support a population specific pattern of selection for the Sorbs. Rather than that, the specific association of *FTO* variants in the third intron with BMI in the Sorbs is more likely to be explained by specific environmental factors interacting with the genetic background in the Sorbs.

Given the fact that the strongest effects on BMI in the Sorbs is on the third intron, rs17818902, we also investigated its potential impact on the transcription factor binding sites. In *silico* analyses using publically available transcription factor databases suggested that the minor rs17818902 allele would predict a novel binding site for TCF3 and that of rs8053740 for SOX17. TCF3 acts as a transcriptional regulator involved in the initiation of neuronal differentiation [51,52] whereas SOX17 is an important player in the regulation of embryonic development and in the determination of the cell fate [53]. However, the causal functional variant remains to be discovered. Thus, studies on pathways downstream of TCF3 may pave the path for better understanding the mechanism underlying associations of FTO with obesity. Nevertheless, it has to be acknowledged that a recent study strongly suggested a direct interaction of noncoding regions in the first intron of *FTO* showed enhancer activity with the promoter of the homeobox gene *IRX3* thus regulating IRX3 expression [54]. However, the clear association connecting functional variants in the first intron and obesity remains vague. Secondly, the experiments of loss of function on *IRX3* were conducted in human cerebellum [55]. In contrast, there is strong evidence for the role of FTO in the complex pathophysiology of obesity (systematically reviewed in [56]). For example, it has been showed that the highest expression of FTO is in the brain region controlling food intake [8] and that hypothalamic-specific manipulation of Fto affects food intake in rats [57].

In conclusion, population genetic analyses revealed balancing signatures of selection at the *FTO* locus with a prominent signal in the third intron, a genomic region with strong association with BMI in the Sorbs. Data provide some evidence supporting evolutionary selective pressure on genes associated with obesity.

## Supporting Information

S1 Table Species included in the PAML analyses. (DOC)

S2 Table *FTO* SNPs included in the analyses. (DOC)

S3 Table Population genetic measures according to Haplotter Phase II. (DOC)

S4 Table Sequences included in transcription factor binding sites analysis. (DOC)

S5 Table Differences in putative transcription factor binding predicted by SNPs in intron three of the *FTO*. (DOC)

S6 Table Functional characteristics of the identified transcription factors. (DOC)

S1 Fig *FTO* intron three with linkage disequilibrium (LD) structure, unstandardized iHS and Fst. (DOC)
