# Supplementary material for: Signatures of Natural Selection at the FTO (Fat Mass and Obesity Associated) Locus in Human Populations
Source: PLoS One. 2015 Feb 3;10(2):e0117093. doi: 10.1371/journal.pone.0117093 (PMC4315420; doi:10.1371/journal.pone.0117093)
Supplement: S1 Table — (DOC) [file pone.0117093.s001.doc]

**Supplemental Table S1: Species included in the PAML analyses**

| Species name | Common | Accession number | Database |
| --- | --- | --- | --- |
| *Anolis carolinensis* | Anole lizard | ENSACAT00000016528 | Ensembl |
| *Bos taurus* | Cattle | ENSBTAT00000016588 | Ensembl |
| *Canis familiaris* | Dog | ENSCAFT00000015044/XM_535301.2 | Ensembl/ NCBI |
| *Cavia porcellus* | Guinea pig | ENSCPOT00000013060 | Ensembl |
| *Choloepus hoffmanni* | Sloth | ENSCHOT00000001256 | Ensembl |
| *Dipodomys ordii* | Ord's kangaroo rat | ENSDORT00000009325 | Ensembl |
| *Echinops telfairi* | Lesser hedgehog tenrec | ENSETET00000012512 | Ensembl |
| *Equus caballus* | Horse | ENSECAT00000024522 | Ensembl |
| *Felis catus* | Cat | ENSFCAT00000005268 | Ensembl |
| *Gorilla gorilla* | Western gorilla | ENSGGOT00000011194 | Ensembl |
| *Homo sapiens* | Human | ENSG00000140718 | Ensembl |
| *Loxodonta africana* | African elephant | ENSLAFT00000015643 | Ensembl |
| *Macaca mulatta* | Rhesus macaque | ENSMMUG00000020583 | Ensembl |
| *Microcebus murinus* | Mouse lemur | ENSMICT00000016851 | Ensembl |
| *Monodelphis domestica* | Opossum | ENSMODT00000012333 | Ensembl |
| *Mus musculus* | Mouse | ENSMUST00000069718 | Ensembl |
| *Myotis lucifugus* | Little brown bat | ENSMLUG00000003855 | Ensembl |
| *Ochotona princeps* | Pika | ENSOPRT00000017284 | Ensembl |
| *Ornithorhynchus anatinus* | Platypus | ENSOANT00000021166 | Ensembl |
| *Oryctolagus cuniculus* | Rabbit | ENSOCUT00000001741 | Ensembl |
| *Otolemur garnettii* | Northern greater galago | ENSOGAT00000005136 | Ensembl |
| *Ovis aries* | Sheep | NM_001104931.1 | NBCI |
| *Pan troglodytes* | Chimpanzee | ENSPTRT00000048611 | Ensembl |
| *Pongo pygmaeus* | Orangutan | ENSPPYT00000008644 | Ensembl |
| *Procavia capensis* | Hyrax | ENSPCAT00000009824 | Ensembl |
| *Pteropus vampyrus* | Large flying fox | ENSPVAT00000011100 | Ensembl |
| *Rattus norvegicus* | Norwegian rat | NM_001039713.1 | NCBI |
| *Sorex araneus* | Common shrew | ENSSART00000002293 | Ensembl |
| *Spermophilus tridecemlineatus* | Thirteen-lined ground squirrel | ENSSTOT00000012696 | Ensembl |
| *Sus scrofa* | Pig | NM_001112692.1 | NCBI |
| *Tarsius syrichta* | Philippine tarsier | ENSTSYT00000008890 | Ensembl |
| *Tupaia belangeri* | Northern tree shrew | ENSTBET00000009717 | Ensembl |
| *Tursiops truncatus* | Common bottlenose dolphin | ENSTTRT00000012713 | Ensembl |
| *Vicugna pacos* | Alpaca | ENSVPAT00000003238 | Ensembl |
| *Xenopus laevis* | African clawed frog | NM_001094012.1 | NCBI |
