# Supplementary material for: Signatures of Natural Selection at the FTO (Fat Mass and Obesity Associated) Locus in Human Populations
Source: PLoS One. 2015 Feb 3;10(2):e0117093. doi: 10.1371/journal.pone.0117093 (PMC4315420; doi:10.1371/journal.pone.0117093)
Supplement: S2 Table — (DOC) [file pone.0117093.s002.doc]

**Supplemental Table S2: *FTO* SNPs included in the analyses**

|  | ID-number | Position (bp) | Region relative to *FTO* |  | ID-number | Position (bp) | Region relative to FTO |
| --- | --- | --- | --- | --- | --- | --- | --- |
| 1 | rs2110844 | 52013417 | 5’ region | 24 | rs1111487 | 52119630 | 5’ region |
| 2 | rs16952242 | 52031751 | 5’ region | 25 | rs4390585 | 52119893 | 5’ region |
| 3 | rs16952252 | 52041481 | 5’ region | 26 | rs10521294 | 52120096 | 5’ region |
| 4 | rs9929873 | 52048177 | 5’ region | 27 | rs16952304 | 52136214 | 5’ region |
| 5 | rs7204496 | 52054099 | 5’ region | 28 | rs2024472 | 52137359 | 5’ region |
| 6 | rs8054299 | 52056156 | 5’ region | 29 | rs7195082 | 52142324 | 5’ region |
| 7 | rs8043918 | 52070534 | 5’ region | 30 | rs7193898 | 52142422 | 5’ region |
| 8 | rs8049033 | 52070556 | 5’ region | 31 | rs4784317 | 52143686 | 5’ region |
| 9 | rs17801498 | 52070953 | 5’ region | 32 | rs8060020 | 52145565 | 5’ region |
| 10 | rs17801966 | 52091302 | 5’ region | 33 | rs12597881 | 52153867 | 5’ region |
| 11 | rs3809634 | 52095658 | 5’ region | 34 | rs6499628 | 52161678 | 5’ region |
| 12 | rs7186754 | 52097010 | 5’ region | 35 | rs1544667 | 52162031 | 5’ region |
| 13 | rs3095635 | 52098599 | 5’ region | 36 | rs2111121 | 52167552 | 5’ region |
| 14 | rs3095634 | 52098891 | 5’ region | 37 | rs12928335 | 52172247 | 5’ region |
| 15 | rs3095633 | 52098979 | 5’ region | 38 | rs16952362 | 52195389 | 5’ region |
| 16 | rs3095571 | 52102494 | 5’ region | 39 | rs3213758 | 52196939 | 5’ region |
| 17 | rs3095631 | 52103301 | 5’ region | 40 | rs9302649 | 52200325 | 5’ region |
| 18 | rs17802269 | 52107643 | 5’ region | 41 | rs17803830 | 52200361 | 5’ region |
| 19 | rs17194040 | 52108399 | 5’ region | 42 | rs8052792 | 52201746 | 5’ region |
| 20 | rs7190220 | 52109683 | 5’ region | 43 | rs9940797 | 52205469 | 5’ region |
| 21 | rs8051156 | 52112725 | 5’ region | 44 | rs9922369 | 52205983 | 5’ region |
| 22 | rs11863156 | 52113591 | 5’ region | 45 | rs13329973 | 52218367 | 5’ region |
| 23 | rs17838423 | 52117231 | 5’ region | 46 | rs1078620 | 52222098 | 5’ region |
| 47 | rs2111119 | 52229255 | 5’ region | 72 | rs9922047 | 52363781 | FTO/Intron1 |
| 48 | rs12597471 | 52235930 | 5’ region | 73 | rs16952522 | 52364999 | FTO/Intron1 |
| 49 | rs16952410 | 52237696 | 5’ region | 74 | rs17817288 | 52365265 | FTO/Intron1 |
| 50 | rs12051119 | 52238931 | 5’ region | 75 | rs1477196 | 52365759 | FTO/Intron1 |
| 51 | rs17215517 | 52241086 | 5’ region | 76 | rs1121980 | 52366748 | FTO/Intron1 |
| 52 | rs7192060 | 52249700 | 5’ region | 77 | rs7193144 | 52368187 | FTO/Intron1 |
| 53 | rs8055834 | 52278396 | 5’ region | 78 | rs16945088 | 52370025 | FTO/Intron1 |
| 54 | rs7404301 | 52292884 | 5’ region | 79 | rs8057044 | 52370115 | FTO/Intron1 |
| 55 | rs1421091 | 52297274 | 5’ region | 80 | rs8050136 | 52373776 | FTO/Intron1 |
| 56 | rs7203521 | 52326794 | FTO/Intron1 | 81 | rs9939609 | 52378028 | FTO/Intron1 |
| 57 | rs16952479 | 52328079 | FTO/Intron1 | 82 | rs9931164 | 52382739 | FTO/Intron1 |
| 58 | rs8048396 | 52328250 | FTO/Intron1 | 83 | rs9941349 | 52382989 | FTO/Intron1 |
| 59 | rs16952482 | 52329084 | FTO/Intron1 | 84 | rs9930506 | 52387966 | FTO/Intron1 |
| 60 | rs4396532 | 52330548 | FTO/Intron1 | 85 | rs2111650 | 52390317 | FTO/Intron1 |
| 61 | rs9933611 | 52331386 | FTO/Intron1 | 86 | rs6499646 | 52401034 | FTO/Intron1 |
| 62 | rs7186637 | 52337603 | FTO/Intron1 | 87 | rs17218700 | 52402080 | FTO Gene |
| 63 | rs1861869 | 52347682 | FTO/Intron1 | 88 | rs9935403 | 52404427 | FTO Gene |
| 64 | rs1861868 | 52347903 | FTO/Intron1 | 89 | rs11075994 | 52407580 | FTO Gene |
| 65 | rs17217144 | 52348263 | FTO/Intron1 | 90 | rs1421090 | 52407671 | FTO Gene |
| 66 | rs9940700 | 52352910 | FTO/Intron1 | 91 | rs9972717 | 52408805 | FTO Gene |
| 67 | rs13334933 | 52353137 | FTO/Intron1 | 92 | rs11075996 | 52415525 | FTO Gene |
| 68 | rs12446228 | 52357888 | FTO/Intron1 | 93 | rs2042032 | 52416084 | FTO Gene |
| 69 | rs9939973 | 52358069 | FTO/Intron1 | 94 | rs10852522 | 52416278 | FTO Gene |
| 70 | rs9940128 | 52358255 | FTO/Intron1 | 95 | rs7204916 | 52421901 | FTO/Intron 3 |
| 71 | rs1421085 | 52358455 | FTO/Intron1 | 96 | rs10521308 | 52422627 | FTO/Intron 3 |
| 97 | rs12921970 | 52422727 | FTO/Intron 3 | 122 | rs9937234 | 52495551 | FTO Gene |
| 98 | rs16952577 | 52425817 | FTO/Intron 3 | 123 | rs8056040 | 52499645 | FTO Gene |
| 99 | rs17818902 | 52429307 | FTO/Intron 3 | 124 | rs4784330 | 52499785 | FTO Gene |
| 100 | rs17818920 | 52429404 | FTO/Intron 3 | 125 | rs12935710 | 52500306 | FTO Gene |
| 101 | rs6499651 | 52431902 | FTO/Intron 3 | 126 | rs12708942 | 52503705 | FTO Gene |
| 102 | rs8053367 | 52432985 | FTO/Intron 3 | 127 | rs1861551 | 52505695 | FTO Gene |
| 103 | rs8053740 | 52433213 | FTO/Intron 3 | 128 | rs9806929 | 52507417 | FTO Gene |
| 104 | rs7203051 | 52433650 | FTO/Intron 3 | 129 | rs1344503 | 52510447 | FTO Gene |
| 105 | rs7205009 | 52433945 | FTO/Intron 3 | 130 | rs16952649 | 52513553 | FTO Gene |
| 106 | rs7205213 | 52434067 | FTO/Intron 3 | 131 | rs12918495 | 52517866 | FTO Gene |
| 107 | rs8061228 | 52439872 | FTO Gene | 132 | rs12932428 | 52518028 | FTO Gene |
| 108 | rs12448529 | 52440158 | FTO Gene | 133 | rs11864972 | 52520564 | FTO Gene |
| 109 | rs11075999 | 52440360 | FTO Gene | 134 | rs7205426 | 52531308 | FTO Gene |
| 110 | rs2111114 | 52440953 | FTO Gene | 135 | rs7205617 | 52531396 | FTO Gene |
| 111 | rs12597422 | 52445239 | FTO Gene | 136 | rs7203181 | 52540981 | FTO Gene |
| 112 | rs9933889 | 52456007 | FTO Gene | 137 | rs12925189 | 52542774 | FTO Gene |
| 113 | rs8060649 | 52463600 | FTO Gene | 138 | rs6499657 | 52546479 | FTO Gene |
| 114 | rs10521304 | 52466158 | FTO Gene | 139 | rs6499658 | 52550205 | FTO Gene |
| 115 | rs10521303 | 52466686 | FTO Gene | 140 | rs11644943 | 52553085 | FTO Gene |
| 116 | rs1362571 | 52469271 | FTO Gene | 141 | rs17823199 | 52556431 | FTO Gene |
| 117 | rs1558756 | 52474009 | FTO Gene | 142 | rs17823223 | 52557139 | FTO Gene |
| 118 | rs9934504 | 52474380 | FTO Gene | 143 | rs1111483 | 52558408 | FTO Gene |
| 119 | rs17820875 | 52484291 | FTO Gene | 144 | rs7194907 | 52560984 | FTO Gene |
| 120 | rs9926180 | 52486108 | FTO Gene | 145 | rs8053888 | 52561306 | FTO Gene |
| 121 | rs2111112 | 52495133 | FTO Gene | 146 | rs9940629 | 52562312 | FTO Gene |
| 147 | rs9932411 | 52562664 | FTO Gene | 172 | rs1861554 | 52607268 | FTO Gene |
| 148 | rs16952725 | 52571768 | FTO Gene | 173 | rs7194243 | 52613660 | FTO Gene |
| 149 | rs16952728 | 52572144 | FTO Gene | 174 | rs17825567 | 52615272 | FTO Gene |
| 150 | rs13337356 | 52574811 | FTO Gene | 175 | rs860713 | 52626966 | FTO Gene |
| 151 | rs12324955 | 52577187 | FTO Gene | 176 | rs2192872 | 52632128 | FTO Gene |
| 152 | rs1125392 | 52577646 | FTO Gene | 177 | rs2689249 | 52639871 | FTO Gene |
| 153 | rs8049235 | 52578510 | FTO Gene | 178 | rs16952906 | 52640668 | FTO Gene |
| 154 | rs4784337 | 52581767 | FTO Gene | 179 | rs11076015 | 52644081 | FTO Gene |
| 155 | rs8056199 | 52582673 | FTO Gene | 180 | rs2540781 | 52645360 | FTO Gene |
| 156 | rs8056502 | 52582815 | FTO Gene | 181 | rs12447427 | 52648090 | FTO Gene |
| 157 | rs11864881 | 52586805 | FTO Gene | 182 | rs1558687 | 52653366 | FTO Gene |
| 158 | rs1861356 | 52591346 | FTO Gene | 183 | rs2689247 | 52654660 | FTO Gene |
| 159 | rs7205987 | 52591646 | FTO Gene | 184 | rs2689246 | 52654678 | FTO Gene |
| 160 | rs13334214 | 52592442 | FTO Gene | 185 | rs2540784 | 52654835 | FTO Gene |
| 161 | rs16952770 | 52592453 | FTO Gene | 186 | rs16952951 | 52656928 | FTO Gene |
| 162 | rs13335453 | 52593744 | FTO Gene | 187 | rs16952955 | 52656970 | FTO Gene |
| 163 | rs7200972 | 52593853 | FTO Gene | 188 | rs2075204 | 52658415 | FTO Gene |
| 164 | rs4784338 | 52595472 | FTO Gene | 189 | rs12600060 | 52658962 | FTO Gene |
| 165 | rs12600130 | 52600824 | FTO Gene | 190 | rs1420318 | 52660267 | FTO Gene |
| 166 | rs17226942 | 52601015 | FTO Gene | 191 | rs2540766 | 52663472 | FTO Gene |
| 167 | rs1345390 | 52602016 | FTO Gene | 192 | rs12149010 | 52665424 | FTO Gene |
| 168 | rs2111118 | 52606654 | FTO Gene | 193 | rs2540769 | 52665463 | FTO Gene |
| 169 | rs17227068 | 52606706 | FTO Gene | 194 | rs2665275 | 52665624 | FTO Gene |
| 170 | rs2111116 | 52606753 | FTO Gene | 195 | rs12599672 | 52673577 | FTO Gene |
| 171 | rs8043737 | 52607166 | FTO Gene | 196 | rs3928987 | 52675012 | FTO Gene |
| 197 | rs1876942 | 52675936 | FTO Gene | 222 | rs12934459 | 52746739 | 3’ region |
| 198 | rs11076017 | 52677885 | FTO Gene | 223 | rs16953154 | 52747518 | 3’ region |
| 199 | rs697769 | 52679248 | FTO Gene | 224 | rs4530133 | 52753690 | 3’ region |
| 200 | rs708254 | 52680890 | FTO Gene | 225 | rs12926239 | 52757628 | 3’ region |
| 201 | rs11863548 | 52681013 | FTO Gene | 226 | rs12447727 | 52757715 | 3’ region |
| 202 | rs708251 | 52682397 | FTO Gene | 227 | rs2542674 | 52763527 | 3’ region |
| 203 | rs2665272 | 52684118 | FTO Gene | 228 | rs2542673 | 52764216 | 3’ region |
| 204 | rs16953047 | 52687671 | FTO Gene | 229 | rs1013170 | 52767948 | 3’ region |
| 205 | rs12927155 | 52691301 | FTO Gene | 230 | rs7190251 | 52768636 | 3’ region |
| 206 | rs12445828 | 52692303 | FTO Gene | 231 | rs836994 | 52771042 | 3’ region |
| 207 | rs2540775 | 52694760 | FTO Gene | 232 | rs2221745 | 52782058 | 3’ region |
| 208 | rs2540776 | 52695363 | FTO Gene | 233 | rs12708944 | 52782242 | 3’ region |
| 209 | rs2689269 | 52696065 | FTO Gene | 234 | rs1510174 | 52783302 | 3’ region |
| 210 | rs12597712 | 52710121 | FTO Gene | 235 | rs836996 | 52783856 | 3’ region |
| 211 | rs2072518 | 52710231 | 3’ region | 236 | rs13338173 | 52784584 | 3’ region |
| 212 | rs2075205 | 52710600 | 3’ region | 237 | rs1912414 | 52792882 | 3’ region |
| 213 | rs16953089 | 52713243 | 3’ region | 238 | rs1912413 | 52792941 | 3’ region |
| 214 | rs8063722 | 52724137 | 3’ region | 239 | rs836987 | 52796240 | 3’ region |
| 215 | rs12924565 | 52726394 | 3’ region | 240 | rs836986 | 52797202 | 3’ region |
| 216 | rs12598390 | 52731786 | 3’ region | 241 | rs11640003 | 52797707 | 3’ region |
| 217 | rs879679 | 52733146 | 3’ region | 242 | rs2665278 | 52800165 | 3’ region |
| 218 | rs2689265 | 52734502 | 3’ region | 243 | rs2665277 | 52800189 | 3’ region |
| 219 | rs7204541 | 52741260 | 3’ region | 244 | rs1397138 | 52801049 | 3’ region |
| 220 | rs872378 | 52741385 | 3’ region | 245 | rs9921416 | 52802255 | 3’ region |
| 221 | rs2015991 | 52741503 | 3’ region | 246 | rs7188172 | 52802421 | 3’ region |
| 247 | rs7196426 | 52806149 | 3’ region | 272 | rs4783833 | 52911981 | 3’ region |
| 248 | rs1876943 | 52808863 | 3’ region | 273 | rs16953344 | 52912670 | 3’ region |
| 249 | rs1543030 | 52809165 | 3’ region | 274 | rs8056104 | 52913102 | 3’ region |
| 250 | rs2102497 | 52812723 | 3’ region | 275 | rs11861062 | 52913124 | 3’ region |
| 251 | rs9930298 | 52813526 | 3’ region | 276 | rs4784362 | 52920107 | 3’ region |
| 252 | rs8057929 | 52818224 | 3’ region | 277 | rs12933995 | 52920155 | 3’ region |
| 253 | rs16953231 | 52820072 | 3’ region | 278 | rs4784364 | 52920289 | 3’ region |
| 254 | rs8060235 | 52823266 | 3’ region | 279 | rs11076028 | 52922819 | 3’ region |
| 255 | rs8052052 | 52833744 | 3’ region | 280 | rs11076029 | 52922848 | 3’ region |
| 256 | rs17254042 | 52836904 | 3’ region | 281 | rs11076031 | 52922993 | 3’ region |
| 257 | rs17175620 | 52838792 | 3’ region | 282 | rs7202735 | 52925464 | 3’ region |
| 258 | rs4423418 | 52843592 | 3’ region | 283 | rs11859377 | 52926175 | 3’ region |
| 259 | rs17254362 | 52846606 | 3’ region | 284 | rs12325064 | 52926959 | 3’ region |
| 260 | rs8061517 | 52859799 | 3’ region | 285 | rs11864160 | 52930364 | 3’ region |
| 261 | rs1112899 | 52866686 | 3’ region | 286 | rs7188799 | 52935935 | 3’ region |
| 262 | rs4461068 | 52867553 | 3’ region | 287 | rs11076032 | 52940785 | 3’ region |
| 263 | rs8053360 | 52871138 | 3’ region | 288 | rs13333404 | 52941076 | 3’ region |
| 264 | rs8057997 | 52885828 | 3’ region | 289 | rs8055411 | 52941624 | 3’ region |
| 265 | rs1868686 | 52886075 | 3’ region | 290 | rs7205326 | 52944701 | 3’ region |
| 266 | rs12929895 | 52888482 | 3’ region | 291 | rs7191827 | 52946171 | 3’ region |
| 267 | rs1530790 | 52898581 | 3’ region | 292 | rs4146345 | 52947131 | 3’ region |
| 268 | rs11639638 | 52898952 | 3’ region | 293 | rs2160295 | 52948376 | 3’ region |
| 269 | rs1562797 | 52900570 | 3’ region | 294 | rs13337115 | 52955014 | 3’ region |
| 270 | rs4784361 | 52904922 | 3’ region | 295 | rs12448816 | 52959597 | 3’ region |
| 271 | rs7199196 | 52907108 | 3’ region | 296 | rs12445707 | 52964055 | 3’ region |
| 297 | rs2388636 | 52964753 | 3’ region |  |  |  |  |
| 298 | rs748815 | 52965305 | 3’ region |  |  |  |  |
| 299 | rs12929759 | 52967948 | 3’ region |  |  |  |  |
| 300 | rs12927537 | 52971280 | 3’ region |  |  |  |  |
| 301 | rs4784376 | 52971785 | 3’ region |  |  |  |  |
| 302 | rs1420294 | 52982472 | 3’ region |  |  |  |  |
| 303 | rs1420297 | 52985907 | 3’ region |  |  |  |  |
| 304 | rs7206026 | 52987359 | 3’ region |  |  |  |  |
| 305 | rs4783840 | 52988164 | 3’ region |  |  |  |  |
| 306 | rs7194372 | 52994431 | 3’ region |  |  |  |  |
| 307 | rs1420304 | 52997859 | 3’ region |  |  |  |  |

Close related individuals who shared Individual Identity by descent/IBD > 0.05 were removed. 307 SNPs from 34 individuals are included according to: minor allele frequency (MAF) > 0.01, Hardy-Weinberg equilibrium (HWE) > 0.0001, missingness per SNP < 0.05, and missingness per sample < 0.07.
