# Supplementary material for: Signatures of Natural Selection at the FTO (Fat Mass and Obesity Associated) Locus in Human Populations
Source: PLoS One. 2015 Feb 3;10(2):e0117093. doi: 10.1371/journal.pone.0117093 (PMC4315420; doi:10.1371/journal.pone.0117093)
Supplement: S3 Table — (DOC) [file pone.0117093.s003.doc]

**Supplemental Table S3: Population genetic measures according to Haplotter Phase II**

|  |  |  | CEU | | ASN | | YRI | | Fst | | |
| --- | --- | --- | --- | --- | --- | --- | --- | --- | --- | --- | --- |
| SNP | *p* | Intron | iHS | d a f | iHS | d a f | iHS | d a f | CEU vs. YRI | CEU vs ASN | ASN vs. YRI |
| rs1861869 | 0.01465 | 1 | -0.692 | 0.533 | -0.454 | 0.167 | 0.820 | 0.367 | 0.028 | 0.150 | 0.052 |
| rs1861868 | 0.00938 | 1 | -0.920 | 0.500 | -0.713 | 0.156 | 0.375 | 0.183 | 0.111 | 0.137 | 0.001 |
| rs9940700 | 0.07348 | 1 | 1.959 | 0.208 | -0.518 | 0.322 | -0.164 | 0.725 | 0.268 | 0.016 | 0.156 |
| rs9939973 | 0.005447 | 1 | -1.254 | 0.475 | 0.966 | 0.206 | -0.772 | 0.467 | 0.000 | 0.081 | 0.076 |
| rs9940128 | 0.01583 | 1 | -1.262 | 0.475 | 0.966 | 0.206 | -0.772 | 0.467 | 0.000 | 0.081 | 0.076 |
| rs9922047 | 0.01035 | 1 | 1.075 | 0.417 | -0.168 | 0.356 | 0.979 | 0.150 | 0.088 | 0.004 | 0.051 |
| rs16952522 | 0.1976 | 1 | n. a. | 0.008 | -0.424 | 0.083 | n. a. | n. a. | n. a. | 0.027 | n. a. |
| rs17817288 | 0.006795 | 1 | -0.383 | 0.558 | 0.418 | 0.583 | 0.956 | 0.433 | 0.016 | 0.001 | 0.022 |
| rs1477196 | 0.02183 | 1 | -0.027 | 0.283 | -0.907 | 0.289 | n. a. | 0.033 | 0.117 | 0.000 | 0.103 |
| rs1121980 | 0.004003 | 1 | 1.866 | 0.525 | -0.152 | 0.794 | 1.261 | 0.525 | 0.000 | 0.081 | 0.081 |
| rs7193144 | 0.003387 | 1 | 2.140 | 0.558 | 0.642 | 0.856 | 1.370 | 0.583 | 0.001 | 0.109 | 0.094 |
| rs16945088 | 0.5796 | 1 | 1.295 | 0.108 | 0.326 | 0.100 | 0.101 | 0.367 | 0.092 | 0.000 | 0.104 |
| rs8050136 | 0.003092 | 1 | 2.058 | 0.550 | 0.716 | 0.856 | 1.120 | 0.533 | 0.000 | 0.115 | 0.125 |
| rs9939609 | 0.008727 | 1 | 1.991 | 0.550 | 0.707 | 0.483 | 0.464 | 0.856 | 0.004 | 0.115 | 0.160 |
| rs9930506 | 0.02465 | 1 | 1.645 | 0.525 | 0.128 | 0.789 | -1.051 | 0.817 | 0.096 | 0.077 | 0.001 |
| rs11075994 | 0.9537 | 2 | 0.567 | 0.642 | 0.087 | 0.844 | -0.434 | 0.892 | 0.087 | 0.055 | 0.005 |
| rs1421090 | 0.1254 | 2 | 0.533 | 0.275 | -0.248 | 0.562 | 1.104 | 0.317 | 0.056 | 0.077 | 0.002 |
| rs9972717 | 0.7354 | 2 | -0.479 | 0.125 | n. a. | 0.033 | n. a. | n. a. | n. a. | 0.031 | n. a. |
| rs10852522 | 0.001739 | 2 | 0.706 | 0.575 | -0.326 | 0.728 | 0.410 | 0.633 | 0.004 | 0.025 | 0.010 |
| rs10521308 | 0.7355 | 3 | n. a. | 0.025 | -1.434 | 0.267 | 0.149 | 0.108 | 0.028 | 0.099 | 0.037 |
| rs17818902 | 0.000632 | 3 | 0.923 | 0.706 | 0.396 | 0.822 | 0.667 | 0.592 | 0.013 | 0.020 | 0.065 |
| rs17818920 | 0.0007213 | 3 | 0.923 | 0.706 | 0.396 | 0.822 | 0.667 | 0.592 | 0.013 | 0.020 | 0.065 |
| rs8053367 | 0.0009839 | 3 | 0.252 | 0.558 | -0.077 | 0.472 | 0.332 | 0.442 | 0.014 | 0.007 | 0.001 |
| rs8053740 | 0.001025 | 3 | 0.252 | 0.558 | -0.077 | 0.472 | 0.332 | 0.442 | 0.014 | 0.007 | 0.001 |
| rs7203051 | 0.001116 | 3 | 0.252 | 0.558 | -0.077 | 0.472 | 0.435 | 0.450 | 0.012 | 0.007 | 0.000 |
| rs7205009 | 0.001319 | 3 | 0.252 | 0.558 | -0.077 | 0.472 | 0.349 | 0.442 | 0.014 | 0.007 | 0.001 |
| rs7205213 | 0.001292 | 3 | 0.252 | 0.558 | -0.073 | 0.450 | 0.349 | 0.442 | 0.014 | 0.011 | 0.000 |

iHS=integrated Haplotype score; d a f=derived allele frequency; CEU=Central Europeans; ASN=Asians; YRI=Yoruba from Ibadan; n. a.=not available
