# Supplementary material for: Signatures of Natural Selection at the FTO (Fat Mass and Obesity Associated) Locus in Human Populations
Source: PLoS One. 2015 Feb 3;10(2):e0117093. doi: 10.1371/journal.pone.0117093 (PMC4315420; doi:10.1371/journal.pone.0117093)
Supplement: S4 Table — (DOC) [file pone.0117093.s004.doc]

**Supplemental Table S4:** **Sequences included in transcription factor binding sites analysis**

| **SNP, minor < major allele** | **Sequence** |
| --- | --- |
| rs10521308, A < G | GGAGTATTCATAAAAACGCT [A/G] TGATGTTTAATGTTATCTTA |
| rs17818902, G < T | GTCAGTAGTCTCAGTGTAAG [G/T] TCTAGATTCTTTCCTATGGT |
| rs17818920, C < A | CATGCCATTCATGTGACACT [C/A] TTGCAGCTGGGAATTGATTG |
| rs8053367, T < G | CATGAACTTGGCTTTCTGAT [T/G] TGTTCATTTTCACCATGCCT |
| rs8053740, C < G | GGGAAAACATGCTTTATTGT [C/G] CATTTTGAAAGCTTTGCATC |
| rs7203051, C < G | CTAGTACAGCAGTTTAAAGT [C/G] TGATGGAATTTCAATTTAAA |
| rs7205009, T < C | ACTCTTGTACCCCAGAAAAA [T/C] TAGTCTTGAGTATCCTTCCA |
| rs7205213, T < C | TGAGTATCCTTCCAGAAATA [T/C] TATATGTGCTTGATATTTAT |

Each variant is flanked by 20bp upstream and downstream, respectively. All sequences are from UCSC Genome Browser on Human May 2004 (NCBI35/hg17). Prediction of transcription factor binding sites was performed for sequences with major or minor alleles separately.
