# Supplementary material for: Signatures of Natural Selection at the FTO (Fat Mass and Obesity Associated) Locus in Human Populations
Source: PLoS One. 2015 Feb 3;10(2):e0117093. doi: 10.1371/journal.pone.0117093 (PMC4315420; doi:10.1371/journal.pone.0117093)
Supplement: S5 Table — (DOC) [file pone.0117093.s005.doc]

**Supplemental Table S5: Differences in putative transcription factor binding sites predicted by SNPs in intron three of the *FTO***

| **SNP, minor < major allele** | **Reference sequence**  **major allele** | **Reference sequence**  **minor allele** |
| --- | --- | --- |
| rs10521308, A<G | - | - |
| rs17818902, G<T | - | TCF3 |
| rs17818920, C<A | HLF, SOX17 | - |
| rs8053367, T<G | Irf-1 | FREAC-2, HNF-3beta, HFH-1, HFH-2, |
| rs8053740, C<G | - | SOX17 |
| rs7203051, C<G | - | - |
| rs7205009, T<C | - | - |
| rs7205213, T<C | [HNF-3beta](http://asp.ii.uib.no:8090/cgi-bin/CONSITE/jaspartf?ID=MA0047&jobID=13814796322091) | - |

Transcription factor binding site are presented for sequences containing either minor or major alleles. All analyses are standardized to the forward strand.
