# Supplementary material for: Signatures of Natural Selection at the FTO (Fat Mass and Obesity Associated) Locus in Human Populations
Source: PLoS One. 2015 Feb 3;10(2):e0117093. doi: 10.1371/journal.pone.0117093 (PMC4315420; doi:10.1371/journal.pone.0117093)
Supplement: S6 Table — (DOC) [file pone.0117093.s006.doc]

**Supplemental Table S6: Functional characteristics of the identified transcription factors**

| **Transcription factor , alias**  **(full name)** | **Function description** |
| --- | --- |
| FOXA2, HNF-3beta (Forkhead Box A2) | FOXA2 plays a role in controlling the expression of genes which are important for the maintenance of beta-cell glucose sensing and glucose homeostasis [1]. It negatively regulates the basal transcription and expression of the human *FT*O gene [2]. FOXA2 has been linked to sporadic cases of maturity-onset diabetes of the young [3]. |
| *FOXD3, HFH-2 (Forkhead box D3)* | FOXD3 acts as a transcriptional repressor [4]. It is involved in the segregation of the neural crest lineage from the neuro-epithelium as well as represses melanogenesis [5]. |
| FOXF2, FREAC-2 (Forkhead Box F2) | FOXF2 is expressed in lung and placenta, which has been shown to transcriptionally activate several lung-specific genes [6, 7]. |
| *FOXQ1, HFH-1* (Forkhead Box Q1) | FOXQ1is involved in embryonic development, cell cycle regulation, tissue-specific gene expression, cell signaling, and tumorigenesis [8]. |
| *HLF* (Hepatic Leukemia Factor) | This gene encodes a member of the proline and acidic-rich (PAR) protein family, a subset of the bZIP transcription factors. The encoded protein forms homodimers or heterodimers with other PAR family members and binds sequence-specific promoter elements to activate transcription [9]. |
| IRF1, Irf-1 (Interferon Regulatory Factor 1) | It encodes interferon regulatory factor 1 and is a transcription activator [10]. It is responsible in regulating apoptosis and tumor-suppression [11]. |
| SOX17 (Sry-related high-mobility group box 17) | Regulation of embryonic development and in the determination of the cell fate [12,13]. |
| TCF3, Thing-E47* (Transcription factor 3) | Transcriptional regulator. Involved in the initiation of neuronal differentiation. [14,15,[16] |

*Transcription factor name updated tracking <http://snpper.chip.org/mapper-pages/MA0092.html>

Supplemental References

1. Wang H, Gauthier BR, Hagenfeldt-Johansson KA, Iezzi M, Wollheim CB (2002) Foxa2 (HNF3beta ) controls multiple genes implicated in metabolism-secretion coupling of glucose-induced insulin release. J. Biol. Chem. 277 (20): 17564–17570.

2. Guo J, Ren W, Ding Y, Li A, Jia L et al. (2012) Fat mass and obesity associated gene (FTO) expression is regulated negatively by the transcription factor Foxa2. PLoS ONE 7 (12): e51082.

3. Hinokio Y, Horikawa Y, Furuta H, Cox NJ, Iwasaki N et al. (2000) Beta-cell transcription factors and diabetes: no evidence for diabetes-associated mutations in the hepatocyte nuclear factor-3beta gene (HNF3B) in Japanese patients with maturity-onset diabetes of the young. Diabetes 49 (2): 302–305.

4. Yaklichkin S, Steiner AB, Lu Q, Kessler DS (2007) FoxD3 and Grg4 physically interact to repress transcription and induce mesoderm in Xenopus. J. Biol. Chem. 282 (4): 2548–2557.

5. Kos R, Reedy MV, Johnson RL, Erickson CA (2001) The winged-helix transcription factor FoxD3 is important for establishing the neural crest lineage and repressing melanogenesis in avian embryos. Development 128 (8): 1467–1479.

6. Pierrou S, Hellqvist M, Samuelsson L, Enerbäck S, Carlsson P (1994) Cloning and characterization of seven human forkhead proteins: binding site specificity and DNA bending. EMBO J. 13 (20): 5002–5012.

7. Hellqvist M, Mahlapuu M, Samuelsson L, Enerbäck S, Carlsson P (1996) Differential activation of lung-specific genes by two forkhead proteins, FREAC-1 and FREAC-2. J. Biol. Chem. 271 (8): 4482–4490.

8. Bieller A, Pasche B, Frank S, Gläser B, Kunz J et al. (2001) Isolation and characterization of the human forkhead gene FOXQ1. DNA Cell Biol. 20 (9): 555–561.

9. Inaba T, Roberts WM, Shapiro LH, Jolly KW, Raimondi SC et al. (1992) Fusion of the leucine zipper gene HLF to the E2A gene in human acute B-lineage leukemia. Science 257 (5069): 531–534.

10. Itoh S, Harada H, Nakamura Y, White R, Taniguchi T (1991) Assignment of the human interferon regulatory factor-1 (IRF1) gene to chromosome 5q23-q31. Genomics 10 (4): 1097–1099.

11. Tanaka N, Ishihara M, Kitagawa M, Harada H, Kimura T et al. (1994) Cellular commitment to oncogene-induced transformation or apoptosis is dependent on the transcription factor IRF-1. Cell 77 (6): 829–839. Available: http://www.sciencedirect.com/science/article/pii/0092867494901325.

12. Katoh M (2002) Molecular cloning and characterization of human SOX17. Int J Mol Med 9 (2): 153–157.

13. Niakan KK, Ji H, Maehr R, Vokes SA, Rodolfa KT et al. (2010) Sox17 promotes differentiation in mouse embryonic stem cells by directly regulating extraembryonic gene expression and indirectly antagonizing self-renewal. Genes & Development 24 (3): 312–326.

14. Bain G, Maandag EC, Izon DJ, Amsen D, Kruisbeek AM et al. (1994) E2A proteins are required for proper B cell development and initiation of immunoglobulin gene rearrangements. Cell 79 (5): 885–892.

15. Yi F, Pereira L, Hoffman JA, Shy BR, Yuen CM et al. (2011) Opposing effects of Tcf3 and Tcf1 control Wnt stimulation of embryonic stem cell self-renewal. Nat Cell Biol 13 (7): 762–770.

16. Pereira L, Yi F, Merrill BJ (2006) Repression of Nanog Gene Transcription by Tcf3 Limits Embryonic Stem Cell Self-Renewal. Molecular and Cellular Biology 26 (20): 7479–7491.
