# Supplementary material for: Signatures of Natural Selection at the FTO (Fat Mass and Obesity Associated) Locus in Human Populations
Source: PLoS One. 2015 Feb 3;10(2):e0117093. doi: 10.1371/journal.pone.0117093 (PMC4315420; doi:10.1371/journal.pone.0117093)
Supplement: S1 Fig — (DOC) [file pone.0117093.s007.doc]

**Supplemental Figure S1: *FTO* intron three with linkage disequilibrium (LD) structure,** **unstandardized iHS and Fst.**


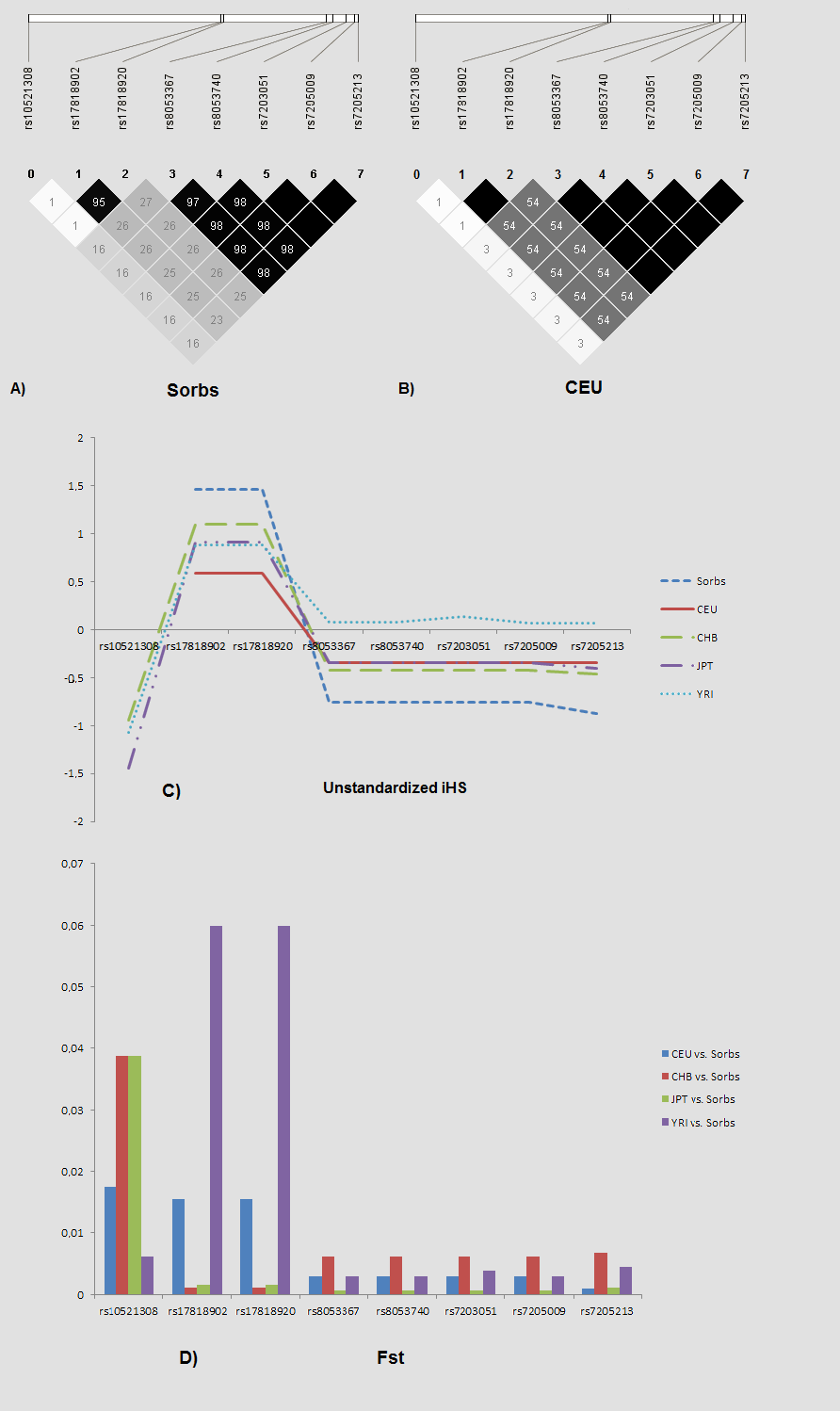


LD is presented as r^2^ within Sorbs A) and CEU B), respectively. Values in the figure indicate r^2^, where r^2^ greater than 1 is not displayed. Unstandardized iHS values in intron three among populations are showed on C), none of iHS > |1.5|, indicating no signs of selection. D) population differences in terms of Fst values and the comparisons conducted between each population (CEU,CHB,JPT and YRI) with Sorbs.
